# A sodium ion conducting gel polymer electrolyte with counterbalance between 1-ethyl-3-methylimidazolium tetrafluoroborate and tetra ethylene glycol dimethyl ether for electrochemical applications

**DOI:** 10.1039/d4ra01615g

**Published:** 2024-04-30

**Authors:** Maitri Patel, Kuldeep Mishra, N. A. Chaudhary, Vaishali Madhani, J. J. Chaudhari, Deepak Kumar

**Affiliations:** a Gujarat Technological University Ahmedabad Gujarat-382424 India deepak.kumar06@gov.in fwtdrdeepakkumar@gmail.com; b Vishwakarma Government Engineering College Ahmedabad Gujarat-382424 India; c Symbiosis Institute of Technology (SIT), Symbiosis International (Deemed University) (SIU) Pune-412115 India; d Department of Applied Physics, Faculty of Technology and Engineering, The Maharaja Sayajirao University of Baroda Vadodara Gujarat-390002 India; e Department of Applied Sciences (Physis), Parul University Vadodara Gujarat-391760 India; f Regional Institute of Education Mysuru, National Council of Educational Research and Training Mysuru-570006 Karnataka India

## Abstract

For sodium batteries, the development of gel polymer electrolytes (GPEs) with remarkable electrochemical properties is in its early stage and persists to be a challenge. In this report we have synthesized a series of GPEs containing a poly(vinyllidene fluoride-*co*-hexafluoropropylene) (PVdF-HFP) and poly (methyl methacrylate) (PMMA) as blend polymer, sodium perchlorate (NaClO_4_) as ion-conducting salt and 1-ethyl-3-methylimidazolium tetrafluoroborate (EMIM-BF_4_) and tetra ethylene glycol dimethyl ether (TEGDME) as molecular solvents. The counter balance between EMIM-BF_4_ and TEGDME is maintained by the electrolyte, which is formed through the optimal weight ratio of 2 : 1. GPEs have an advantageous set of properties, including stability window of 5 V, Na^+^ transference number of 0.20, and a room-temperature ionic conductivity of 5.8 × 10^−3^ S cm^−1^. According to enthalpy and entropy calculations, optimized GPE yields the highest amount of disorder or amorphicity and contributes to greatest conductivity. XRD analysis supports this argument. Thermal investigations show that optimized GPE may preserve gel phase up to 125 °C. The prototype sodium cell fabricated with optimize GPE has a specific capacity of 281 mA h g^−1^ and open circuit voltage of 2.5 V. The optimized GPE exhibits potential for future electrochemical applications.

## Abstract 2

For sodium batteries, the development of gel polymer electrolytes (GPEs) with remarkable electrochemical properties is in its early stage and persists to be a challenge.

## Graphical abstract

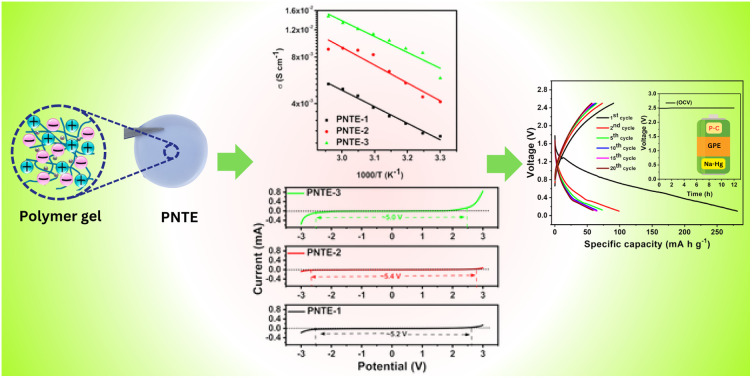

## 1. Introduction

Rechargeable Na-based batteries have become the subject of renewed attention in the last couple of years for their potential utilizations, due to their economical nature.^1^ Owing to its uniform dispersal throughout the Earth's shell, sodium is regarded as an element that is abundant. Furthermore, sodium has an electrochemical similarity to lithium and a low redox potential (0.3 V higher than lithium). As a result, lithium-based batteries can provide valuable insights for growth of Na batteries.^2^ Na-based batteries similar to lithium–ion batteries comprise an anode, cathode, and electrolyte. In order to fabricate and design Na–ion batteries with high efficiency, it is imperative to consider appropriate anode materials, cathode materials, electrolytes, and separators.^3^ The primary focus of research over the past decade has been the development of sodium-based batteries through the utilization of various cathode materials and architectures. In batteries, the performance characteristics are significantly influenced by electrolytes, in addition to electrodes.^4^ With the emergence of innovative electrode constituents, electrolyte's demands will change, necessitating specialized research efforts to mature extremely efficient electrolyte structures. Cost-effective, highly conductive, effortlessly plentiful; ecologically sound, and electrically reliable electrolytes are essential for Na-based batteries.^5^ Due to their exceptional ion-conduction properties, transport number, and inherent ionic conductivity, liquid electrolytes are extensively utilized in electrochemical applications.^6,7^ Electrolytes in liquid form are susceptible to leakage, short-circuiting, flammability, and poor thermal stability.^5^ Solid polymer electrolytes (SPEs) offer distinct benefits over liquid electrolytes (LEs), including reduced dendritic growth, less leakage, and enhanced thermal stability.^8^ SPEs are, nevertheless, afflicted with interfacial instability, a diminished discharge capacity, and high resistance at the electrolyte/electrode interface.^9^ In recent times, a significant number of the aforementioned challenges have been resolved through the substitution of LEs and SPEs with gel polymer electrolytes (GPEs). These GPEs are formed by immobilizing liquid electrolytes (*e.g.*, ionic salt dissolved in polar organic solvent) within a suitable polymer. GPEs are highly desirable for use in electrochemical application owing to their electrochemical stability, favorable thermal stability, and flexible characteristics. Additionally, their ionic conductivity is approximately 10^−3^ S cm^−1^ at *RT*. Furthermore, GPE serves as a separator and an electrolyte both.^10,11^ For use in electrochemical application, people around the world are preoccupied with the formulation of novel flexible GPE films exhibiting high ionic conductivity and other desirable physical and electrochemical properties.^12^

Molecular solvents play a vital role in the GPEs. By decreasing crystallinity of polymer, plasticizer induces formation of an increased number of dissociated free cations and anions *via* the separation of ion pairs. Conductivity is exclusively observed in the amorphous region. Consequently, as the degree of amorphicity of the GPE increases, so its conductivity will also boost by inclusion of solvents.^13^ In addition to their stability and reduced flammability, electrolytes containing TEGDME feature high conductivity.^14^ It has many other advantageous characteristics, including a high boiling point and minimal volatility.^15^ Ionic liquids (ILs) have garnered significant attention in recent investigations owing to their distinctive physical and chemical characteristics, including incombustibility, miscibility with various organic solvents, and non-volatility. Due to their broad potential window and high ionic conductivity, ILs are viable electrolytes from an electrochemical aspect. ILs have garnered considerable curiosity in recognition of their promising utility in electrochemical capacitors and batteries.^16–18^ Numerous studies demonstrate the application of ionic liquid and glyme as molecular solvents for fabrication of GPEs for lithium and sodium based electrochemical application. Syali *et al.* reported cocktail GPEs by utilizing carbonate, glyme and ionic liquid molecular solvents for Na based batteries and EDLC application.^19^ Wu *et al.* designed GPEs with the combination of *N*-methoxyethyl-*N*-methylpyrrolidinium bis(trifluorome-thanesulfonyl)-imide (P_1,2O1_TFSI) and TEGDME for application in lithium–sulfur battery.^20^ Rao *et al.* fabricated Li–S cell by utilizing a GPE containing *N*-methyl-*N*-butylpiperidinium bis(trifluoromethanesulfonyl) imide (PPR_14_TFSI) and poly (ethylene glycol) dimethyl ether (PEGDME).^21^ Wu *et al.* synthesized Li–S battery by employing binary salt based GPE with Nmethoxyethyl-*N*-methylpyrrolidinium bis(trifluoromethanesulfonyl)-imide (Pyr_1,2O1_TFSI) TEGDME in ratio of 7 : 3.^22^ Song *et al.* reported polymer electrolyte by utilizing 1-ethyl-3-methylimidazolium bis(fluorosulfonyl)imide and SiO_2_ nanoparticles with PEO matrix for Na metal batteries.^23^

Scientists are making efforts to discover a sodium–ion conducting gel polymer electrolyte that is commercially viable. Even though there are numerous reports on the development of GPEs, the authors have not found any report on the counterbalance between the solvents in order to advance the development of electrolytes for Na batteries. This study aims to address this gap found in the literature. By combining EMIM-BF_4_ and TEGDME, we proposed a novel ionic liquid-based GPE system for electrochemical application in this study that aims to amplify electrochemical properties of GPEs. The ether group present in ionic liquid confers a comparatively high level of conductivity. Ethers may serve as viable alternatives for co-solvents, thereby potentially enhancing the conductivity of GPEs. These properties are critical for optimizing the capacity and rate performance of any batteries.^24^ Although ethers with low molecular weights, such as diethylene glycol dimethyl ether (DEGDME) and 1,2-dimethoxyethane (DME), have a low viscosity, their low boiling and flashing points render them incompatible with safety regulations. TEGDME is chosen in the electrolyte without significantly compromising the intrinsic safety of the ionic liquid. Consequently, we investigate the possibility for a counterbalance between the TEGDME and the ether-functionalized ionic liquid in order to advance the development of Na batteries that exhibit commendable safety attributes.^20^ In various mass ratios, we utilized binary mixture consisting of 2 M NaClO_4_ + EMIM-BF_4_/TEGDME. The electrolyte mixes were analyzed for their physical and electrochemical characteristics. The optimal ratio for balancing the composition of EMIM-BF_4_/TEGDME mixed electrolytes was identified. Additionally, we examined the electrochemical behavior of Na based prototype cell utilizing the optimized electrolyte mixtures.

## 2. Experimental

### 2.1 Materials

As the host polymer, PVdF-HFP with avg. molecular weight 400 000, NaClO_4_ (98% purity), tetraethylene glycol dimethyl ether (TEGDME) (>99.00%), *N*-methyl pyrrolidone (NMP) (99.5%), conducting carbon black (99+% metal basis) and phosphorus red (≥97.0%) were procured from Sigma-Aldrich. PMMA with a molecular weight of 350 000 was acquired from Alfa Aesar. The EMIM-BF_4_ extrapure, catalysis, and nanotechnology grade chemical was obtained from SRL and utilized without further processing.

### 2.2 Fabrication of gel polymer electrolyte (GPE)

**Fig. 1:**
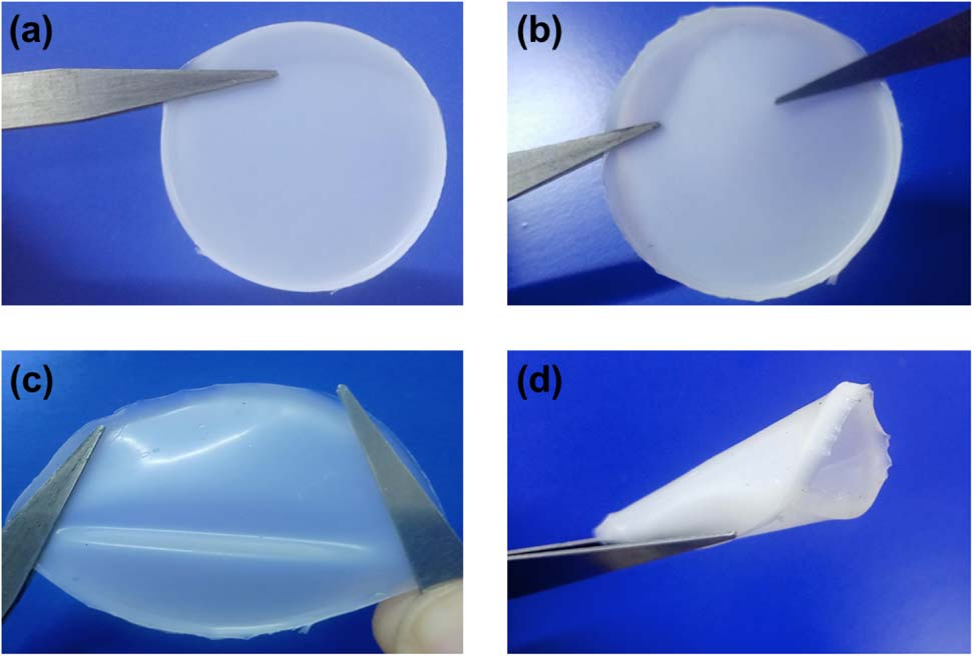
The images of PNTE-3 (a **and** b) free-standing, (c) stretching and (d) folding.

The GPE was synthesized with solvent casting approach. PVdF-HFP and PMMA in 4 : 1 ratio was dissolved in Acetone and stirred homogeneously for 12 h. The mixture of PVdF-HFP/PMMA matrix was mixed with 2 M NaClO_4_ in EMIMBF_4_ + TEGDME (EMIMBF_4_ : TEGDME 1 : 2, 1 : 1, and 2 : 1 by wt.) for 5 h to synthesize GPEs. Following that, the acetone-containing homogeneous solutions containing all the constituents were emptied in Petri dishes. The acetone was then permitted to evaporate at *RT* in order to acquire GPE, which were dimensionally stable and free-standing as shown in Fig. 1.

The composition and sample codes of the GPEs are detailed in Table 1.

**Table 1:** Sample code and their composition

Sample code	Composition
PNTE-1	PVdF-HFP/PMMA + EMIMBF_4_ : TEGDME (1 : 2) + NaClO_4_
PNTE-2	PVdF-HFP/PMMA + EMIMBF_4_ : TEGDME (1 : 1) + NaClO_4_
PNTE-3	PVdF-HFP/PMMA + EMIMBF_4_ : TEGDME (2 : 1) + NaClO_4_

### 2.3 Preparation of electrodes and fabrication of sodium cell

In order to synthesize the cathode for the sodium cell, the solid-state route was utilized. The components of the electrode-conductive carbon black, amorphous red phosphorus, and optimized GPE namely PNTE-3 (which was employed as the binder) were blended with a mortar and pestle in a weight ratio of 7 : 2 : 1. After approximately 10 h of thorough blending of the aforementioned composite, a consistent electrode slurry formed in NMP (10 g mL^−1^). The conclusive electrode was manufactured by micropipette-casting of the electrode slurry onto aluminum current collectors (1 cm^2^), which were then subjected to vacuum drying at 80 °C for a duration of 12 h. As the anode material for the prototype sodium battery, sodium-mercury amalgam (Na–Hg) was utilized. In order to design a prototype sodium battery, an electrolyte sample comprising the optimized GPE (PNTE-3) with optimal conductivity was installed between a fabricated cathode and Na–Hg amalgam.

### 2.4 Instrumentation

Impedance measurements were conducted by employing Impedance Analyzer, Hioki IM3536 LCR Meter, Japan; throughout frequencies from 4 Hz to 8 MHz and temperatures between 30 and 65 °C with scan-rate of 10 mV. Electrolyte films in a square shape were placed between two stainless steel electrodes to determine AC impedance. To measure the electrochemical stability window and to confirm moment of Na ion the cyclic voltammetry study has been performed with a scan rate 5 mV S^−1^ by utilizing SS, Na–Hg reversible electrode. The total ion transport number (*t*_ion_) was obtained using chronoamperometry with SS electrode at 0.75 V utilizing dc polarisation technique. Sodium ion transport number (*t*_Na^+^_) was obtained by utilizing Bruce–Vincent methodology and 20 mV voltage with Na–Hg reversible electrode. CV, *t*_ion_, and *t*_Na^+^_ number investigations are done upon electrochemical workstation, CHI 660E, USA. DCS of polymer gel systems was conducted using TA instruments, model: Q100 with a heating rate, 10 °C min^−1^ in a nitrogen atmosphere, ranging from −80 to 200 °C. The TGA was conducted through a PerkinElmer TGA7 instrument, which heated the sample from *RT* to 600 °C in a nitrogen air at a rate, 10 °C min^−1^. FTIR studies were performed using a PerkinElmer Frontier FTIR spectrometer with a resolution of 4 cm at wavenumbers ranging from 700 to 4000 cm^−1^ with 32 scan per sample. The synthesised films' X-ray diffraction (XRD) patterns were captured utilizing Rigaku smart lab diffractometer equipped with Cu Kα radiation spanning a Bragg angle (2*θ*) range of 5–65° with a scan rate of 4° min^−1^. All electrochemical measurements on prototype sodium cell such as open circuit potential and galvanostatic charge–discharge (GCD) were conducted by employing Zive SP1 electrochemical workstation, WonATech Co., Ltd, Korea.

## 3. Results and discussions

### 3.1 Ionic conductivity measurement

**Fig. 2:**
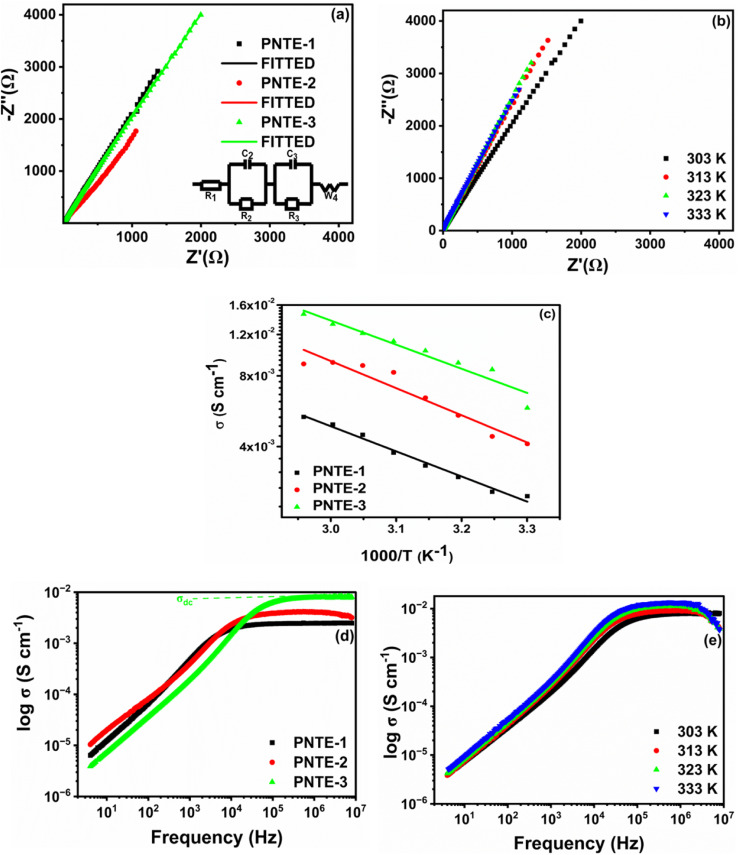
(a) Nyquist curve (*Z*′ *versus Z*″) at *RT* for PNTE-1,2,3 (equivalent circuit of impedance data: (inset)) (b) Nyquist curve (*Z*′ *versus Z*″) at various temperature for PNTE-3 (c) variation of *σ* with temperature for PNTE-1,2,3 (d) *σ*_ac_*vs.* frequency at *RT* for PNTE-1,2,3 (e) *σ*_ac_*vs.* frequency at various temperature for PNTE-3.

EIS is utilized to assess ionic conductivity (*σ*) of produced GPEs. Fig. 2(a) illustrates Nyquist plot of GPEs that were developed at *RT*. In the absence of the characteristic semicircles in higher-frequency area, these curves displayed evident oblique straight lines in low-frequency zone, signifying that conduction of GPEs was merely due to ion migration as a result of the system's facile ion mobility. The real axis intercept values (*R*_b_) of high-frequency impedance data on the Cole–Cole plot are about 19.66 Ω for PNTE-1, 10.10 Ω for PNTE-2 and approximately 6.69 Ω PNTE-3 sample.

Fig. 2(b) illustrates Nyquist curves of optimized GPEs at 30, 40, 50 and 60 °C. *R*_b_ drops as temperature rises in GPE (Fig. 2(b)). The *R*_b_ is correlated with *σ* of GPE according to the subsequent relationship.1
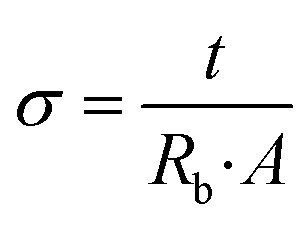
where, symbols have their usual meanings. The *σ* of GPE namely PNTE-1, computed using eqn (1) is around 2.4 × 10^−3^ S cm^−1^, lesser than that of PNTE-2 and PNTE-3 which are 4.1 × 10^−3^ S cm^−1^ and 5.8 × 10^−3^ S cm^−1^ respectively. The enhanced *σ* of the PNTE-3 is due to the more amorphous nature of blending of PVdF-HFP with PMMA host, which offers enough free space for ion movement *via* enhanced segmental mobility and increased liquid phase content. Experimental data points for complex impedance of various GPEs at *RT* are revealed in Fig. 2(a). The equivalent circuit for fitted data of complex impedance is depicted in Fig. 2(a) inset.

The components of equivalent circuit of the cell have been determined using the EC-Lab software. Eqn (2) is used to fit complex impedance experimental data points into Randles Cell Circuit model, which is given in Fig. 2(a).2
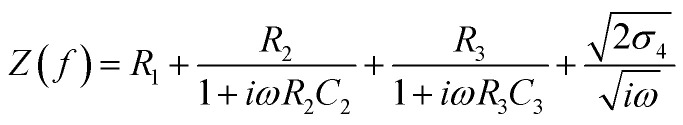
where *R*_1_ represents the cell's resistance, the 2nd term indicates electrode polarization, the 3rd term indicates conduction phenomena, and *σ*_4_ indicates Warburg element, which is corresponding to the Warburg impedance (W_4_) in the Randles cell circuit model. The Warburg element is used to interpret the mechanism of the diffusion process in low frequency zone. The parameters obtained from fitting and equivalent circuit model are listed in Table 2. Multiple relaxation behaviors were observed, leading to the calculation of individual relaxation timings, including the electric double layer formation time constant (*τ*_EDL_) and the conduction relaxation time constant (*τ*_*σ*_) for each sample, as outlined in eqn (3) and (4) respectively. These parameters offer insights into the electrical properties and relaxation mechanisms within the GPEs samples. From Table 2 it can be noticeable that *R*_b_ decreases with decline in *τ*_EDL_ and *τ*_*σ*_ value.

3 *τ*_EDL_ = *R*_2_*C*_2_

4 *τ*_*σ*_ = *R*_3_*C*_3_

**Table 2:** EIS model circuit parameters for sample at *RT*

Sample code	*R*_b_	*R*_1_ (Ω)	*C*_2_ (µF)	*R*_2_ (Ω)	*C*_3_ (µF)	*R*_3_ (Ω)	*σ*_4_ (Ω s^−1/2^)	*τ*_EDL_ (s)	*τ*_*σ*_ (s)
PNTE-1	19.66	18.19	10.11	229.1	11.61	7385	1332	0.002316	0.08574
PNTE-2	10.10	8.875	8.018	197.2	18.46	3324	1270	0.001581	0.06136
PNTE-3	6.69	4.316	4.183	236.6	7.169	8017	1269	0.000990	0.05747

Fig. 2(c) demonstrates the variation in log *σ* in relation to the reciprocal of the absolute temperature for GPEs with various concentrations of TEGDME and EMIMBF_4_ in temperature range 30 to 65 °C. Fig. 2(c) indicates that conductivity of the systems does not have a sudden increase with temperature, suggesting that GPEs have a fully amorphous structure.^25^ It is apparent from the graph that *σ* of each sample rises with increasing temperature, which is consistent with Arrhenius relation. The correlation between temperature and conductivity may be attributed to a reduction in viscosity, which consequently enhances the flexibility of the chain.^26^ This phenomenon can be elucidated by considering free volume model and the hopping of charge carriers between localized states.^27^ Polymer ionizes salt into anions and cations upon exposure to an applied electric field and temperature. By transitioning between localized states, these ions increase the ionic conductivity. When the temperature rises, a segment's vibrational energy becomes strong enough to overcome the hydrostatic pressure exerted by the atoms around it, creating a little area around its own volume where vibrational motion is possible. Consequently, an increase in conductivity is brought about by free volume around polymer, which also induces ion and polymer segment mobility. Because there is more free volume at a higher temperature, conductivity increases. A larger free volume is also provided in the polymer electrolyte system by rising amorphous content with temperature.^28^ Throughout the entire temperature range, the temperature-dependent ionic conductivity of PNTE-3 is greater than other counterpart GPEs of this study.

The thermodynamic parameters are crucial for understanding the temperature-dependent conductivity of polymer electrolytes include the change in activation energy (Δ*E*_a_), enthalpy of activation (Δ*H*), and entropy of activation (Δ*S*). These parameters are determined through experimental analysis by employing an Arrhenius-type relationship^29,30^, which establishes a correlation between conductivity and temperature. The experimental data utilized in this study indicates that *σ* of GPEs upsurges with temperature, suggesting a process of heat activation within the material. This phenomenon can be elucidated through the Arrhenius relationship, which correlates conductivity (*σ*) with temperature (*T*) as follows:5
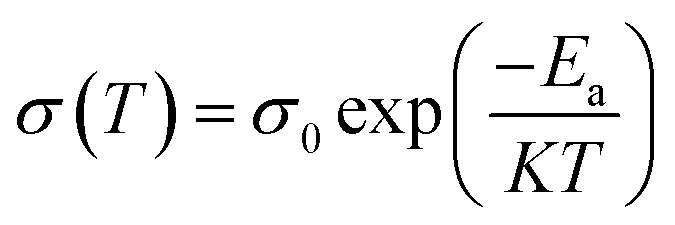
where, symbols have their usual meaning. This suggests heat activation of material has occurred.^31^ Furthermore, if conductivity arises from a thermal activation process, Δ*H* and Δ*S* can be discovered from Eyring relation. The Eyring equation establishes a linear relation obtained by plotting in ln(*σh*/*K*_B_*T*) *vs.* 1000/*T*, given by eqn (6).6
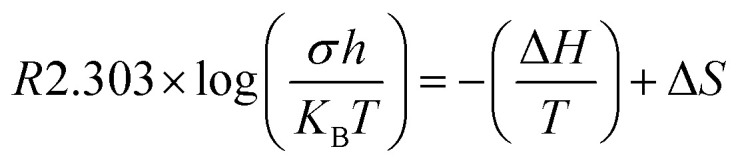
where, symbols have their usual meanings.

The Eyring–Polanyi equation provides a general linear form of the Arrhenius-like appearance by related conductivity to Gibb's function and calculating Δ*H* and Δ*S* is specified by eqn (7).7
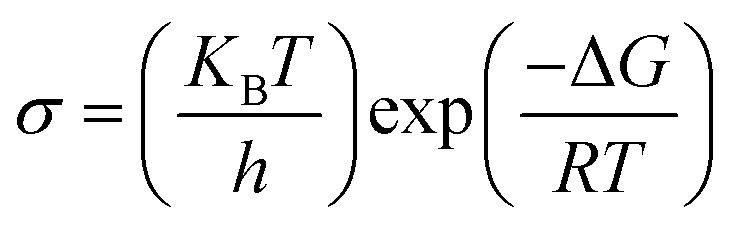
where *R* is the ideal gas constant.

The change in activation enthalpy (Δ*H*) and entropy (Δ*S*) is also expressed as^32^8
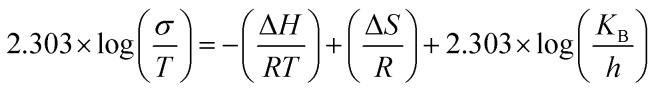


This can be written in the form of *y* = *mx* + *c* as given by eqn (8).

Where slope determines enthalpy of activation, (
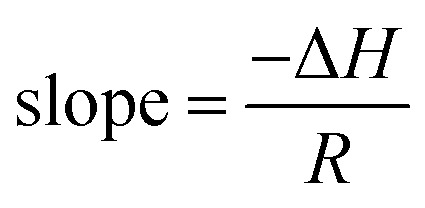
) and intercept supply entropy, Δ*S* [intercept = (Δ*S*/*R*) + ln(*k*/*h*)].

Table 3 shows ionic conductivity, activation energy, enthalpy and entropy values for fabricated GPEs along with their sample code. Δ*H* and Δ*S* are obtained from linear plots of 1000/*T vs.* ln(*σ*/*T*). The enthalpy values obtained for samples PNTE-1, PNTE-2 and PNTE-3 are 23.06, 18.23 and 17.80 J mol^−1^ K^−1^. So, enthalpy value is decreased in GPE sample PNTE-3. The entropy values are about of −214.83, −226.75 and −236.66 J mol^−1^ K^−1^ for PNTE-1, PNTE-2 and PNTE-3 respectively. The optimized GPEs sample PNTE-3 carries the maximum conductivity and lowest activation energy. The lowest enthalpy and highest entropy of GPE PNTE-3 indicate that the GPE carry maximum amorphicity contributing to the faster polymer segmental motion and hence faster ionic mobility.

**Table 3:** Ionic conductivity (*σ*), activation energy (*E*_a_), enthalpy (Δ*H*) and entropy (Δ*S*) values obtained from PNTE series at room temperature

Sample	Ionic conductivity (*σ*) (S cm^−1^)	Activation energy (*E*_a_) (kJ mol^−1^)	Enthalpy (Δ*H*) (J mol^−1^ K^−1^)	Entropy (Δ*S*) (J mol^−1^ K^−1^)
PNTE-1	2.4 × 10^−3^	22.08	23.06	−214.83
PNTE-2	4.1 × 10^−3^	20.46	18.23	−226.75
PNTE-3	5.8 × 10^−3^	19.63	17.80	−236.66

Fig. 2(d) displays the plot of ac conductivity against frequency for GPEs namely PNTE-1, PNTE-2 and PNTE-3. At lower frequencies, the alternating current signal is active for longer periods, leading to anticipated polarizing effects at interface between the electrolyte and electrode. Lower values of ac conductivity indicate that polarization effects are more prominent at lower frequencies. At the electrolyte/electrode interface, polarizing effects will occur due to the increased duration of the ac signal at low frequencies. At lower frequency values, polarization effects predominate, as indicated by the lower values of ac conductivity (*σ*_ac_). A decrease in polarizing effects is observed as the frequency increases, leading to an increase in conductivity values. The *σ*_ac_ patterns begin to flatten for frequencies greater than 10^3^ Hz; consequently. This means that *σ* values are identical across the whole plateau area. This flat zone indicates ionic transport occurs by hopping, and observed *σ* values in this zone are identical to *σ* values obtained using eqn (1). *σ*_ac_ values for optimized GPE (PNTE-3) rise with temperature, but the overall behavior stays constant over the whole frequency range (Fig. 2(e)). Hence, throughout the operational temperature range of the GPE, hopping behaviour is observed at high frequencies and polarizing effects are observed at low frequencies.

### 3.2 Dielectric and modulus studies

Studying the dielectric properties of GPEs helps in comprehending the conductive features of electrolyte systems. The temperature-dependent dielectric behavior was analyzed using the dielectric function. The formula for the frequency dependent complex dielectric permittivity (ε*) is as follows:

9 ε* = *ε*′ − *jε*″

where, symbols have their usual meanings.

By utilizing the dielectric constant (*ε*′), one can examine ion conduction and relaxation mechanisms of GPEs system. The *ε*′of GPEs influences its polarization. The dielectric constant (*ε*′) of an electrolyte system represents the capacity for electric charge storage and dipole alignment per unit volume. It can be mathematically represented as follows:10
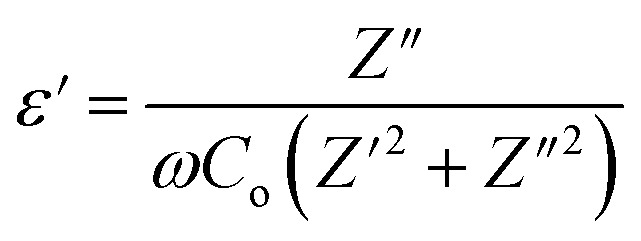
where, symbols have their usual meanings.

**Fig. 3:**
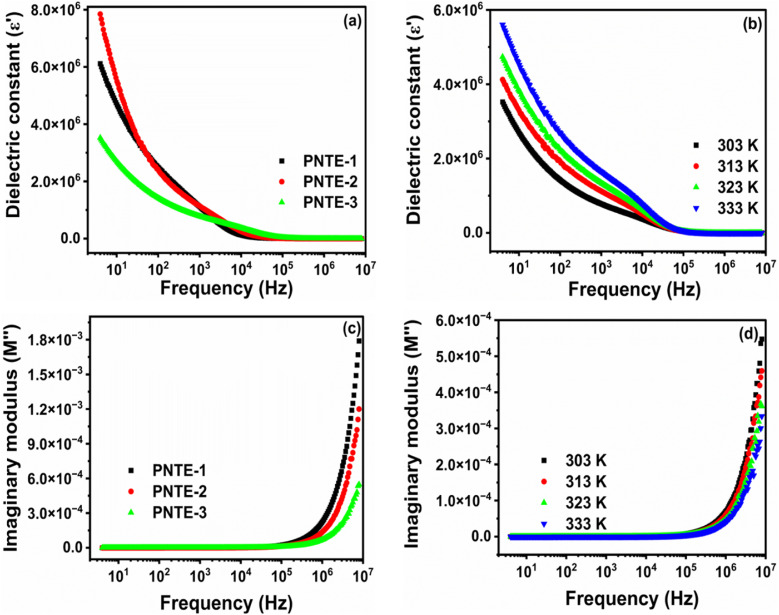
(a) *ε*′ *vs.* frequency at *RT* for PNTE-1,2,3 (b) *ε*′ *vs.* frequency at various temperature for PNTE-3 (c) *M*″ *vs.* frequency at *RT* for PNTE-1,2,3 (d) *M*″ *vs.* frequency at various temperature for PNTE-3.

The dielectric constant of all GPEs system at *RT* is illustrated in Fig. 3(a). Additionally, Fig. 3(b) illustrates the frequency dependence of the dielectric constant of PNTE-3 across different temperatures.

Fig. 3(a) shows the change in *ε*′ for all the samples at *RT*. The plot of *ε*′ shows high values at lower frequencies due to the very higher interfacial capacitance and the buildup of ionic charge at the electrode-GPEs interface. At low frequencies, the system has great energy storage capabilities and experiences maximum energy loss. The reduction in *ε*′ with increasing frequency demonstrates the polar character of the system. When the applied field direction is reversed at high frequency, the charges are unable to keep up, leading to a drop in polarization and a fall in the *ε*′ value.^33^

At temperatures higher than room temperature, dipoles can readily align, although highly cross-linked materials may make orientation challenging at times. Fig. 3(b) shows the changes in *ε*′ of PNTE-3 sample at different temperatures. It is evident that as the frequency increases, the *ε*′ declines steadily and reaches a plateau at high frequencies after a certain point. The increased *ε*′ at high temperatures in low frequency is due to polarization of the electrode and space charge.^34^

Electric modulus assessments (*M*″) have been carried out to differentiate electrode polarization and other interfacial characteristics. Analysis on modulus is helpful in discovering materials that have similar resistances but varying capacitances.^35,36^ The *M*″ may be mathematically characterized as:11
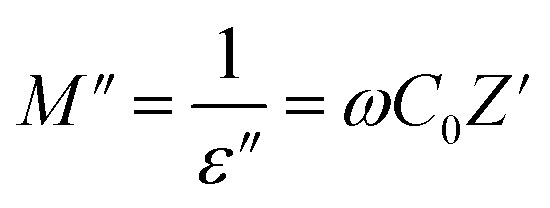
where, symbols have their usual meanings.^37^

Fig. 3(c) illustrates alteration in *M*″ *vs.* frequency at *RT* for GPEs: PNTE-1, PNTE-2, and PNTE-3. A negligible response, close to zero, is seen in low frequency owing to electrode/electrolyte interfacial interaction. This observation further indicates the extensive migration of ions over long distances and the slight impact of polarization effects in small frequency. The existence of an elongated tail in area of lower-frequency indicates that electrodes possess a substantial capacitance. Fig. 3(c) displays that GPE sample PNTE-3 has the lowest *M*″ in high frequency, when compared to GPE PNTE-1 and PNTE-2. Minor values of *M*″at high frequencies enable elevated *σ* and boosted ion dynamics, as shown by the study.^33^

The graph in Fig. 3(d) illustrates the change in *M*″ *vs.* frequency for PNTE-3 at various temperatures. When temperature goes from 303 K to 333 K, the onset swings to the higher frequency, which shows that relaxation process is happening. Moreover, the shift of this modulus towards higher frequencies with increasing temperature might be attributed to enhanced motion of ions inside tailored GPEs. Fig. 3(d) shows that when the temperature increases, there is a drop in values at higher frequencies. The commencement of peak height lowers with increasing temperature as a result of a reduction in *R*_b_ of GPE. In conclusion, modulus measurements corroborated the findings of ionic conductivity and dielectric studies, showing that electrolyte specimen PNTE-3, has superior conductivity, dielectrics, and modulus values when compared to GPEs specimens PNTE-1 and PNTE-2 respectively.

### 3.3 Cyclic voltammetry and transport number measurements

An electrolyte with a large electrochemical stability window (ESW) is crucial for efficient functioning of electrochemical devices. To investigate ESW of three different GPEs: PNTE-1, PNTE-2, and PNTE-3, cyclic voltammetery (CV) were conducted in range −3 to +3 V at ambient temperature with scan rate 5 mV s^−1^.

**Fig. 4:**
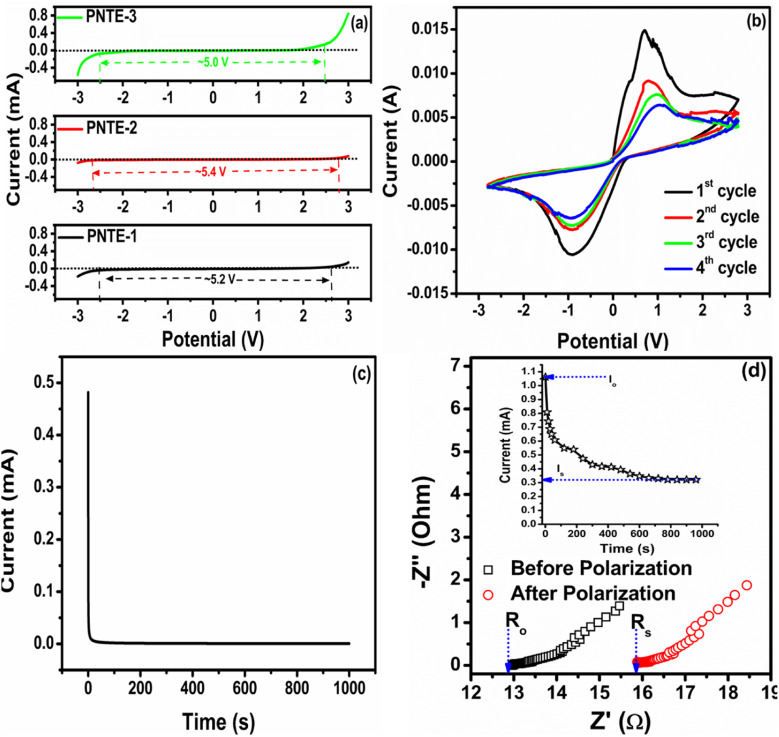
Cyclic voltammetry curvatures (a) with SS/electrolyte/SS for PNTE-1,2,3 (b) with Na–Hg/PNTE-3/Na–Hg (c) *t*_ion_ number for PNTE-3 (d) *t*_Na^+^_ number for PNTE-3.

Fig. 4(a) shows the CV curvatures for prepared GPEs. The electrolyte breakdown voltage, wherein no oxidation or reduction occurs, is determined by the irreversible onset of the current. This value provides insight into the electrochemical stability of the system. The current gradually increases as the voltage across the cells rises. At first, there is a steady flow of electric current through the electrodes. However, once the voltage surpasses a certain threshold, there is a sudden and significant change in the current. This change defines the voltage range within which the membrane operates.^38^ At voltages below 2.5 V, there is a consistent flow of current across the electrodes in all samples. However, as the voltage increases, the current increases gradually, followed by a sudden transition that correlates with the starting point of the electrolyte's decomposition process. This mild current of up to 2.5 V could potentially be ascribed to the altering of the SS surface.^39^ The absence of any adverse reaction and the stability of the GPEs are indicated by the almost negligible current values observed in range of −2.5 V to +2.5 V for all three sample, representing window of approximately 5.2 V, 5.4 V and 5 V for PNTE-1, PNTE-2 and PNTE-3 GPEs respectively, as illustrated in Fig. 4(a). From the standpoint of device application, this operating voltage range is adequate, especially when utilized as the electrolyte in sodium batteries.

Cyclic voltammetry (CV) is used to verify the migration of sodium ions in optimized GPE PNTE-3, using a symmetric reversible cell arrangement, namely Na–Hg|PNTE-3|Na–Hg. The CV plot revealed in Fig. 4(b) displays two peaks in the anodic and cathodic directions, indicating the deposition of sodium ions at the anodic site and the removal of sodium ions at the cathodic site. Therefore, the anodic and cathodic redox processes occur efficiently at the interface between sodium and mercury in the presence of an electrolyte, suggesting the transfer of sodium ions from the anodic site to the cathodic site and *vice versa* when a little voltage is applied. With evidence of sodium ion plating/stripping at the respective electrodes, the CV studies indicate that an ESW of approximately 5 V is satisfactory from an application standpoint for sodium batteries. A multitude of studies have shown analogous sodium–ion migration by using GPEs and a reversible sodium electrode setup.^40,5,41^

The performance of Transference Number Measurement (TNM) relies on characteristics of blocking or non-blocking electrodes. The blocking electrodes selectively allow only electrons to enter and traverse through them, whereas non-blocking electrodes let both electrons and ions (cations or anions) to enter, but only electrons can traverse through them. The determination of TNM may be accomplished through two approaches, namely Wagner's polarization and Bruce–Vincent methods.^42^ In order to determine the type of species that contribute to conductivity in the current electrolyte system, the transport numbers were measured. These numbers provide a quantitative assessment of the extent to which ions and electrons contribute to the overall conductivity. This measurement was done by utilizing Wagner's dc polarization and by applying potential of 0.75 V across the cell. The cell has been polarized using a dc voltage. A decline in electric current over time is seen. The initial high current is due to transfer of ions and electrons. By eliminating ability of ions to travel through the external circuit, the SS electrodes hamper the flow of current solely. As a consequence of the electrode polarization effect, the current diminishes progressively with all-time. Consequently, the observed ultimate constant current can be elucidated exclusively through the electron contribution.^43^ The following expressions are utilized:^36^12
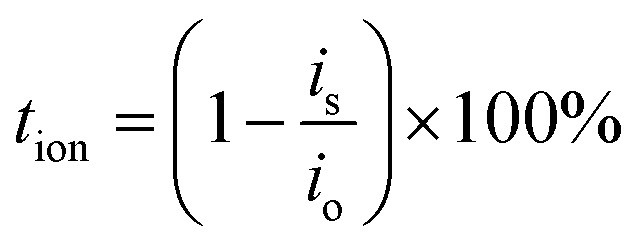

13 *t*_e_ = (1 − *t*_ion_) × 100%

where, *i*_o_ represents initial current and *i*_s_ denotes saturation current obtained from polarization current *versus* time plot.^44^

For the optimal GPE PNTE-3, the time-dependent variation of polarization current is illustrated in Fig. 4(c). It is determined that the value of *t*_ion_ for PNTE-3 sample is 99.08%. More precisely, *t*_e_ is equal to 0.92%. It is evident from this observation that the ion transport number exceeds that of the electron transport number. This observation implies that in the GPE sample that is reported, ions are the predominant species accountable for electrical conduction.^41^

The assessment of the sodium transport number (*t*_Na^+^_) is an essential variable utilized to determine the proportion of sodium ions that contribute to overall *σ* in GPEs. By employing combined ac and dc method, *t*_Na^+^_ of PNTE-3 is computed utilizing the Vincent method. In this methodology, Na–Hg| PNTE-3|Na–Hg cell is polarized for a duration of 2 h through the application of a voltage, Δ*V* = 20 mV. The final and initial currents are subsequently tracked (Fig. 4(d)). Moreover, the cells undergo a.c. impedance measurements both before and after the polarization as a vital aspect of the approach. The electrode–electrolyte contact resistance values are subsequently estimated based on the impedance plots (Fig. 4(d)). The following equation can be utilized to determine sodium–ion transport number (*t*_Na^+^_)14
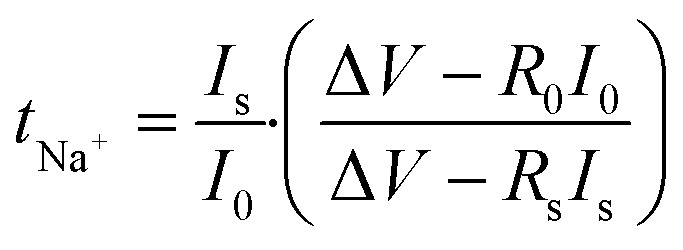
where symbols have their usual meanings.

The *t*_Na^+^_ value for PNTE-3, as determined through eqn (14), is ∼0.20. The observed Na^+^ transport number value in GPE indicate that anionic species play a substantial role in facilitating ion conduction within the electrolyte system.

### 3.4 Thermal studies

**Fig. 5:**
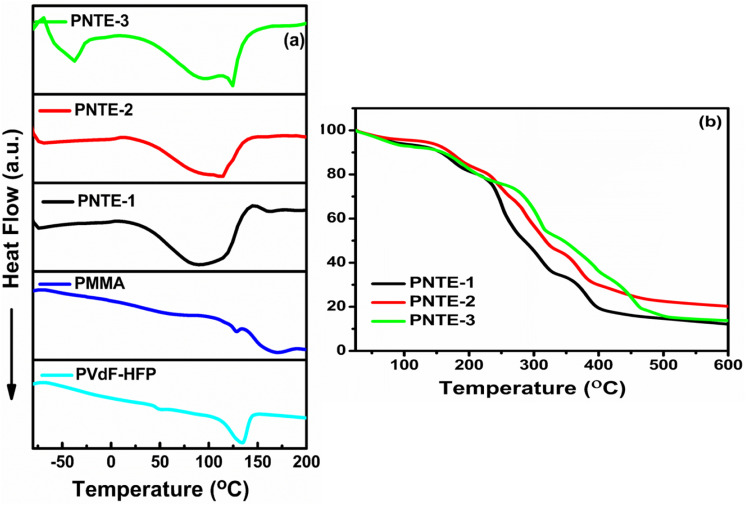
(a) DSC curvatures for PVdF-HFP, PMMA and PNTE-1,2,3 (b) TGA graphs of PNTE-1,2,3.

For usage in electrochemical devices, the constructed GPEs need to be thermally safe. DSC is employed to evaluate thermal stability of the GPEs from −80 °C to 200 °C as depicted in Fig. 5(a). It has been observed that melting temperature (*T*_m_) of PVdF-HFP is 135 °C and glass transition temperature (*T*_g_) of PMMA is 127 °C. After incorporation of liquid electrolyte 2 M NaClO_4_ in EMIMBF_4_ : TEGDME (1 : 2), the endothermic peak is observed at 100 °C (in PNTE-1). In PNTE-2 the peak is observed at 110 °C. The GPE film PNTE-3 is stable up to temperature 125 °C. It is noteworthy that the optimized electrolyte film exhibits featureless DSC curve and maintain stability in the gel-phase across a significantly broad temperature range of −80 to 125 °C. This characteristic renders them highly promising for implementation in electrochemical devices, such as rechargeable sodium batteries.

TGA is done to check thermal stability of GPEs films that were prepared. Fig. 5(b) illustrates the TGA thermograms of prepared GPEs namely PNTE-1, PNTE-2 and PNTE-3 in the temperature range from *RT* to 600 °C. The GPE films composed of PVdF-HFP/PMMA blend decompose in a four-step process, as observed. The initial phase of decomposition was detected at a temperature marginally ∼100 °C, potentially due to the evaporation of surface or residual moisture present in the specimen. The second stage of decomposition occurred between 100–200 °C and is associated with the thermal evaporation of the TEGDME/EMIMBF_4_ solvent and followed by third stage in the range of 200–300 °C due to breakdown of the polymer's main chain. At greater temperatures, mass loss is occurred above 300 °C in fourth stage of decomposition occurred caused by the breakdown of NaClO_4_ salt. The inclusion of NaClO_4_/EMIM-BF_4_/TEGDME in PVdF-HFP/PMMA blending resulted in a reduction in weight loss for the constructed GPEs films, suggesting that the thermal stability of the electrolytes was enhanced and is adequate to support their potential use as electrolyte in electrochemical devices.^45,46^

### 3.5 XRD and FTIR analysis

**Fig. 6:**
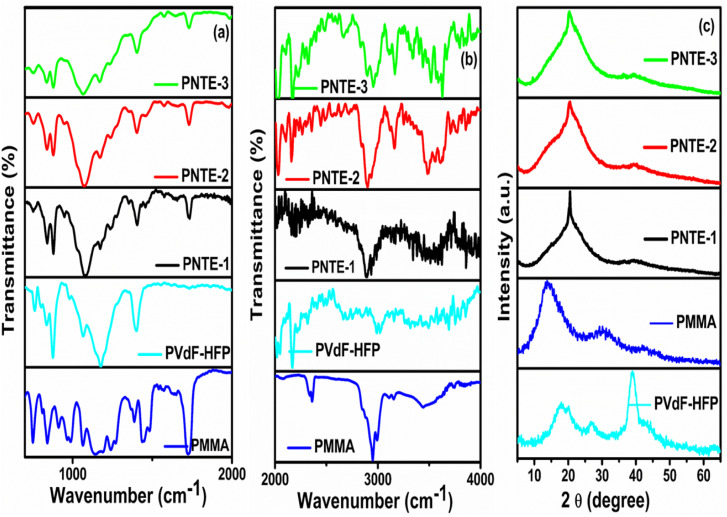
FTIR band for PVdF-HFP, PMMA and PNTE-1,2,3 in wavenumber (a) 700–2000 cm^−1^ and (b) 2000–4000 cm^−1^, and (c) XRD plots for PVdF-HFP, PMMA and PNTE-1,2,3.

Fig. 6(a and b) shows the comparative FTIR plot of pure PMMA, PVdF-HFP and the prepared GPE in the wavenumber region varying from 700–2000 cm^−1^ and 2000–4000 cm^−1^. It was mainly done to observe the interaction of Na^+^ ions, glyme, and ionic liquid (IL) with host polymer PVdF-HFP and PMMA at microscopic level in the designed GPE films. It also provides information on the conformational changes caused by the trapping of liquid electrolytes NaClO_4_/TEGDME/EMIMBF_4_ in the blended polymer matrix.^47^Table 4 shows the wave numbers for PMMA, PVdF, HFP, TEGDME, and EMIMBF_4_.

**Table 4:** List of wave numbers for PMMA, PVdF-HFP, TEGDME and EMIMBF_4_

Materials	IR bands (cm^−1^)	Assignment	Reference
PMMA	747	Rocking deformation vibrations	48
	805	C–O–C symmetric stretching mode	48
	838	Methylene rocking mode	48
	1242	Antisymmetric C–C–O stretch	49
	1386	OCH_3_ deformations	49
	1443	CH_3_ stretching	49
	1726	C=O stretching	50
	2951	CH_3_ asymmetric stretching	50
PVdF-HFP	762	α-Phase	51
	834	γ-Phase	51
	876	Combined CF_2_ and C–C symmetric stretching vibrations and amorphous-HFP	51
	1064	C–C skeletal vibrations and CF_3_ out of plane deformation	52
	1175	antisymmetric CF_2_ stretching	52
	1400	CH_2_ wagging	52
TEGDME	944	Asymmetrical CH_2_ rocking	19
	1242	Asymmetric CH_2_ twisting	19
	1456	CH_2_ scissoring	19
EMIMBF_4_	752	C=C–H out plane bending	19
	1175	Ring in-plane deformation	19
	1572	C–H, C–N (ring) stretching	19

When TEGDME and EMIMBF_4_ are introduced in varying ratios to the blended polymer matrix PMMA : PVdF-HFP, certain absorption bands vanish or shift in the developed samples. There are noticeable changes in the intensity of certain bands. These outcomes definitely show that blended GPEs based on glyme and IL successfully synthesised. The absorption bands detected in 756–698 cm^−1^ of pure PMMA is owed to the presence of bending, ring out of plane or rocking deformation vibrations of PMMA, while band at 762 cm^−1^ in PVdF-HFP polymer corresponds to α-phase of crystalline PVdF. The band at 805, 838 cm^−1^ in pure PMMA polymer is assigned to C–O–C symmetric stretching and methylene rocking. The band 834, 877 cm^−1^ are due to γ-phase of PVdF and combined CF_2_ and C–C symmetric stretching vibrations and amorphous-HFP. The peaks of pure PMMA and PVdF-HFP at 762, 747 cm^−1^ begin to merge and weaken when TEGDME and EMIMBF_4_ have been added to the blend polymer matrix of PMMA : PVdF-HFP. The peak has been shifted to 752 cm^−1^ of C=C–H of EMIMBF_4_. Other characteristic bands at 838 cm^−1^ of PMMA as well as bands at 834 and 876 cm^−1^ is found to be lifted to 836 and 881 cm^−1^. The peak at 949 cm^−1^ in PNTE-1 and PNTE-2 samples corresponds to asymmetrical CH_2_ rocking in TEGDME plasticizer which has disappeared in PNTE-3 sample. The band at 1064 cm^−1^ of PVDF-HFP is of C–C skeletal vibrations and CF_3_ out of plane deformation are getting broadened and lifted to 1069 cm^−1^, specifying interaction between polymers, plasticizers and salt. The absorption band at 1175, 1400 cm^−1^ corresponds to antisymmetric CF_2_ stretching and CH_2_ wagging of PVdF-HFP gets reduced in intensity on addition of TEGDME and EMIMBF_4_. The band at 1175 cm^−1^ links to ring in-plane deformation of IL. The band at 1242 cm^−1^ is allotted to asymmetric CH_2_ twisting of TEGDME. The peaks at 1456, 1572 cm^−1^ corresponds to CH_2_ scissoring and C–H, C–N (ring) stretching of glyme and IL. The PMMA polymer's characteristic peak, which was previously located at 1726 cm^−1^, has now moved to the higher frequency side at 1731 cm^−1^. The addition of plasticizers has also drastically decreased the peak's intensity. The butyl chain of IL (which also causes the polymer backbone stretching) and the imidazolium cation ring of IL are responsible for the C–H stretching vibrations that cause the peaks in the spectral range of 3200−2800 cm^−1^. The peaks found between 3650 and 3000 cm^−1^ indicate the presence of OH and –OOH groups. The peak at 2951 cm^−1^ is owed to presence of CH_3_ asymmetric stretching of methylene group which has been shifted to lower frequency side and has become weak by incorporating TEGDME and EMIMBF_4_. Based on this, the constructed samples consisting PMMA, PVdF-HFP, NaClO_4_, TEGDME, and EMIMBF_4_ exhibit shifting and lack of vibrational bands, suggesting that the polymer, salt, and plasticizer interactions are favourable to form blended GPEs.^48,49,51–55^

In a GPEs, XRD is an effective method to characterize crystalline phase transitions and alterations.^56^ The XRD patterns of (PVdF-HFP: PMMA) and NaClO_4_-based GPE at different TEGDME-EMIMBF_4_ concentrations are shown in Fig. 6(c). The semicrystalline character of PVdF-HFP is revealed by peaks at 2*θ* ∼18.5° and ∼20°, along with small intensity peak at ∼39°. The (100) + (020) and (021) planes of the non-polar crystalline phase of PVdF are represented by peaks on ∼18.5° and ∼39°, respectively. The (110) plane of the polar crystalline phase of PVdF-HFP is reflected by peak on ∼20°.^57^ The XRD peaks that were detected are compared to the standard JCPDS data (Card No. 00-038-1638), which provides confirmation of the creation of the semi-crystalline phase of the PVdF-HFP copolymer.^58^ The pure PMMA pattern has a broad as well, less prominent peak at 2*θ* ∼13.8°, indicating the PMMA film's entire amorphous nature.^57^ The amorphous hump of PMMA at 2*θ* ∼13.8° is found vanished, and the dominant peaks of PVdF-HFP at 2*θ* ∼20° and 39° are reduced in intensity together with the broadening on addition of TEGDME and EMIMBF_4_, indicating a rise in the films' amorphicity (Fig. 6(c)). The aforementioned analyses show that adding TEGDME-EMIMBF_4_ can decrease the polymer membrane's crystallinity, speed up the mobility of the chain segments within the polymer, and increase the pace at which sodium ions migrate.^59^ With XRD analysis, the degree of crystallinity has been computed to determine if the prepared samples' amorphocity has increased or decreased. The pure PVdF-HFP polymer possesses the degree of crystallinity of 34.40%. When TEGDME and EMIMBF_4_ are added to the blended polymer matrix to obtain electrolyte films PNTE-1 and PNTE-2, the degree of crystallinity further reduces to 22.13 and 16.34% respectively. The crystallinity calculations reveals that the electrolyte sample PNTE-3 has the lowest degree of crystallinity of 13.72%, indicating that the presence of higher content of EMIMBF_4_ ionic liquid inculcate more disordering the polymeric structure than other solvent combinations used in PNTE-1 and PNTE-2. Therefore, it should have the most flexible polymeric chain enabling the faster segmental mobility promoting the higher ionic conductivity in the GPE.^60^

### 3.6 Studies on proto-type sodium battery

**Fig. 7:**
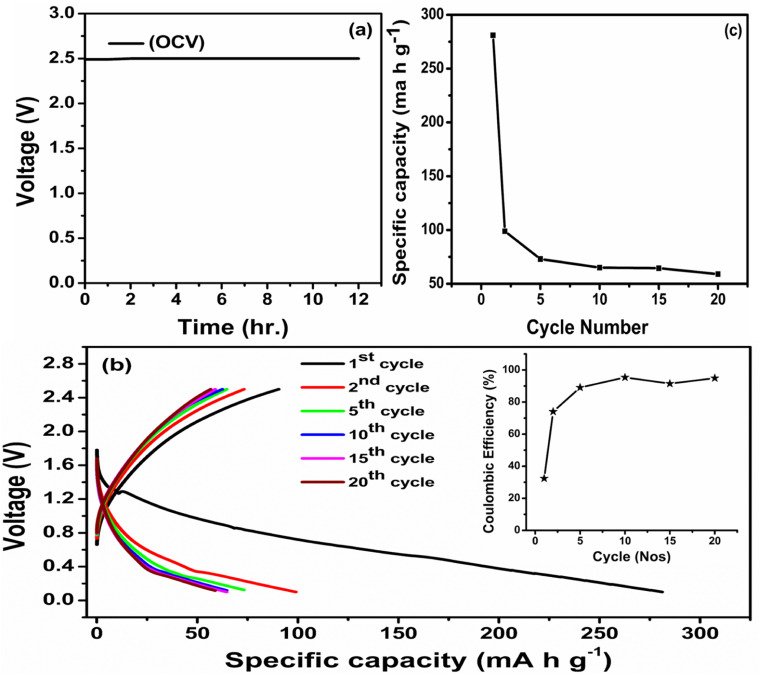
(a) OCV plot for Na cell (b) charge–discharge profile ((inset): coulombic efficiency *vs.* cycle no.) (c) discharge capacity *vs.* cycle number.

In a prototype sodium battery featuring the highest conducting PNTE-3 GPE, a Na–Hg anode, and P–C cathode is fabricated in the cell configuration Na–Hg|PNTE-3|P–C and the performance is studied (Fig. 7). Time-dependent variation of the open-circuit voltage (OCV) of the prototype cell is depicted in Fig. 7(a). The OCV of the cell remain stable at ∼2.5 V throughout the measurement. This confirms the absence of any internal electronic leakage within the cell and favourable chemistry between the PNTE-3 and the electrodes.

The charge–discharge characteristic of the prototype for various cycles under 50 mA g^−1^ current are illustrated in Fig. 7(b). The cell delivers an initial specific discharge capacity of ∼281 mA h g^−1^. The capacity of the cell significantly declines during subsequent charge–discharge cycles, and by the twentieth discharge, it seems to have retained no more than 21.35% of its initial capacity. The two major possible reasons for the above observed capacity deterioration are the formation of passivation layer depositing at the electrolyte/electrode interface at the Na–Hg anode and the inadequate conductivity of the P–C electrode,^61^ which indeed display scope for tailoring electrodes. The coulombic efficiency (CE) of the prototype cell is illustrated in the inset of Fig. 7(b). In this instance, the CE was calculated as the proportion of stripping Na^+^ to plating Na utilized in each cycle.^62^ The initial CE of cell is comparatively low, which is potentially due to the decomposition of NaClO_4_ or the loss of a large amount of sodium due to the formation of solid–electrolyte interface (SEI). Over 20 cycles, the Na–Hg/PNTE-3/P–C maintained a steady coulombic efficiency (CE) of around 94%. As illustrated in Fig. 7(c), the discharge capacity decreases with the number of charge–discharge cycles. During the second discharge, the cell experiences a capacity loss of approximately 35.24%, resulting in a measured capacity of 99 mA h g^−1^. Following the second discharge, the rate of capacity diminishing decelerates, with the discharge capacities of approximately 75, 65, 64.5, and 60 mA h g^−1^ observed for the 5th, 10th, 15th and 20th discharges, respectively.

Although the designed PNTE-3 GPE displays a favourable σ, ESW, and *t*_Na^+^_, the capacity fading of the proto-type sodium cell might be caused by other cell components such as the anode and cathode. The selection of substance for the electrode and the electrolyte/electrode contact are two critical factors in determining the cycling parameters of the cell. During cycling, the GPE may be reduced at the Na–Hg anode and oxidized at the P–C cathode, eventually increasing the size of the passivating solid electrolyte inter-phase (SEI).^63^ This layer may be accountable for consumption of a portion of the cathode's sodium ions, reducing discharge capacity. Another probable explanation is the volume enlargement at the anode portion of the proto-type during cycling, which may increase the charge-transfer resistance at the electrolyte/electrode interface and therefore the internal resistance of the prototype.^64,65^

## 4. Conclusions

In this report we have synthesized a series of GPEs containing a PVdF-HFP and PMMA as polymer-blend matrix, NaClO_4_ as salt and EMIM-BF_4_ and TEGDME as solvents for electrochemical application. GPE with 2 M NaClO_4_ in EMIM-BF_4_/TEGDME (2 : 1) has high ionic conductivity of 5.8 × 10^−3^ S cm^−1^ at *RT* and stability window of 5 V with decent Na^+^ moment features. According to enthalpy and entropy calculations, the optimal GPE yields the highest amount of disorder or amorphicity and contributes with the greatest conductivity. XRD analysis supports this argument. Thermal investigations show that manufactured GPEs may preserve the gel phase up to 125 °C. FTIR spectra show changes in structure and ion–polymer interactions. The Sodium battery with optimal GPE had specific capacity about of 281 mA h g^−1^ and open circuit voltage of 2.5 V. A variety of electrochemical devices, including fuel cells, rechargeable sodium batteries, sensors, electrochromic display devices, solar cells, and supercapacitors, can potentially be designed with aforementioned gel polymer electrolytes.

## Author contributions

Maitri Patel: conceptualization, methodology, measurement, sample preparation, writing – original draft, analysis. Kuldeep Mishra: resources, writing – review and editing, conceptualization, supervision. N. A. Chaudhary: experimentation, analysis and manuscript writing. Vaishali Madhani: manuscript writing, methodology. J. J. Chaudhari: characterization, supervision. Deepak Kumar: formal analysis and investigation, conceptualization, writing – review and editing, resources, supervision, funding acquisition.

## Conflicts of interest

The authors declare no conflicts of interest among them.

## Supplementary Material
